# Bortezomib enhances expression of effector molecules in anti-tumor CD8^+^ T lymphocytes by promoting Notch-nuclear factor-κB crosstalk

**DOI:** 10.18632/oncotarget.5857

**Published:** 2015-09-28

**Authors:** Menaka C. Thounaojam, Duafalia F. Dudimah, Samuel T. Pellom, Roman V. Uzhachenko, David P. Carbone, Mikhail M. Dikov, Anil Shanker

**Affiliations:** ^1^ Department of Biochemistry and Cancer Biology, School of Medicine, Meharry Medical College, Nashville, TN, USA; ^2^ Department of Microbiology and Immunology, School of Medicine, Meharry Medical College, Nashville, TN, USA; ^3^ School of Graduate Studies and Research, Meharry Medical College, Nashville, TN, USA; ^4^ Department of Medicine, James Cancer Center, The Ohio State University, Columbus, OH, USA; ^5^ Host-Tumor Interactions Research Program, Vanderbilt-Ingram Cancer Center, Vanderbilt University, Nashville, TN, USA

**Keywords:** Notch signaling, NF-κB, cancer immunosuppression, T cell Immunotherapy, proteasome inhibition, Immunology and Microbiology Section, Immune response, Immunity

## Abstract

The immunosuppressive tumor microenvironment usurps host antitumor immunity by multiple mechanisms including interference with the Notch system, which is important for various metazoan cell fate decisions and hematopoietic cell differentiation and function. We observed that treatment with the proteasome inhibitor bortezomib in mice bearing various solid tumors resulted in an upregulated expression of various Notch signaling components in lymphoid tissues, thereby increasing CD8^+^T-lymphocyte IFNγ secretion and expression of effector molecules, perforin and granzyme B, as well as the T-box transcription factor eomesodermin. Bortezomib also neutralized TGFβ-mediated suppression of IFNγ and granzyme B expression in activated CD8^+^T-cells. Of note, bortezomib reversed tumor-induced downregulation of Notch receptors, Notch1 and Notch2, as well as increased the levels of cleaved Notch intracellular domain (NICD) and downstream targets Hes1 and Hey1 in tumor-draining CD8^+^T-cells. Moreover, bortezomib promoted CD8^+^T-cell nuclear factor-κB (NFκB) activity by increasing the total and phosphorylated levels of the IκB kinase and IκBα as well as the cytoplasmic and nuclear levels of phosphorylated p65. Even when we blocked NFκB activity by Bay-11-7082, or NICD cleavage by γ-secretase inhibitor, bortezomib significantly increased expression of Notch *Hes1* and *Hey1* genes as well as perforin, granzyme B and eomesodermin in activated CD8^+^T-cells. Data suggest that bortezomib can rescue tumor-induced dysfunction of CD8^+^T-cells by its intrinsic stimulatory effects promoting NICD-NFκB crosstalk. These findings provide novel insights on using bortezomib not only as an agent to sensitize tumors to cell death but also to provide lymphocyte-stimulatory effects, thereby overcoming immunosuppressive actions of tumor on anti-tumor T-cell functions.

## INTRODUCTION

Perturbation of the numerous immune regulatory networks by the immunosuppressive tumor microenvironment usurps host antitumor immune responses. Consequently, the management of cancer becomes extremely difficult, thus requiring therapeutic approaches that subvert immune suppression by tumor. The evolutionarily conserved Notch signaling plays a crucial role in several biological processes such as embryogenesis, cell proliferation, apoptosis, as well as immune cell differentiation and function [1–7]. Dysregulation of Notch signaling can thus lead to several hematological diseases [8–15]. Notch signaling is triggered by receptor-ligand interactions between juxtaposed neighboring cells. The sequential proteolytic cleavage of the Notch intracellular domain (NICD) by A-Disintegrin-And-Metalloproteinase (ADAM) at the S2 site followed by the cleavage at the S3 site by γ-secretase releases NICD, which then translocates to the nucleus for interaction with the transcriptional regulator *RBP-J*κ, otherwise known as *CSL* (“CBF-1, Suppressor of Hairless, Lag-2,” after its mammalian, *Drosophila*, and *Caenorhabditis elegans* orthologues) to initiate transcription of its target genes such as *Hes1* and *Hey1* [16–18]. Moreover, NICD can interact with nuclear factor-κB (NFκB) in T cells by competing with IκBα, thus facilitating NFκB retention in the nucleus [19]. Evidence is emerging to support a functional crosstalk between Notch and NFκB signaling pathways in T cells [20–22]. Notch and NFκB activities are critical for the maturation of CD4^+^CD8^+^ thymocytes [23, 24]. It has also been reported that an increase in NFκB p65 protein levels enhances Notch-mediated activation of the *Hes1* promoter [25]. However, interaction between both signaling pathways is intriguing since NFκB can exert antagonistic or synergistic effects depending on the context of other cellular communication pathways.

We reported that tumor downregulates the crucial Notch signaling to suppress T cell effector function [26]. Recently, we observed that treatment with the proteasome inhibitor bortezomib (BZB) in mice bearing different types of solid tumors results in increased CD8^+^ T lymphocyte IFNγ secretion and expression of the effector molecules perforin and granzyme B as well as the transcription factor eomesodermin by modulating Notch signaling. Bortezomib is an FDA-approved drug for the treatment of multiple myeloma and mantle cell lymphoma [27–34], and we showed that it can sensitize various mouse and human solid tumor cells to apoptosis by upregulating caspase-8 activity [35, 36]. However, the effects of bortezomib on various immune functions are not clear. Both immune stimulatory and suppressive effects of bortezomib on immune cells have been cited [37–44]. Using murine renal and mammary solid tumors expressing a low-avidity model antigen hemagglutinin (HA) [45], or a lung fibrosarcoma expressing human Ras and mutant human p53 as xenogeneic antigens [46], we investigated the effects of bortezomib on the lymphocyte expression of activation and effector molecules during an endogenous anti-tumor T cell response in tumor-bearing mice and explored their underlying mechanisms.

Results show that bortezomib can rescue tumor-induced downregulation of Notch in lymphocytes while enhancing their immune effector function. In particular, bortezomib administration in tumor-bearing mice promoted expression of T cell activation and effector molecules. These bortezomib-mediated T cell effects were associated with increased crosstalk between Notch and NFκB signaling pathways important for T cell cytolytic function. Findings suggest that bortezomib, in addition to its established role in sensitizing tumors to cell death, can provide T cell stimulatory effects. Therapeutic restoration of lymphocytic Notch signaling and effector function could enhance anti-tumor immune surveillance following a carefully optimized bortezomib regimen, which could break tumor resistance to cytolysis and overcome tumor-associated immunosuppression.

## RESULTS

### Bortezomib treatment enhances the expression of CD8^+^ T cell activation and effector molecules in tumor-bearing mice

**Figure 1:**
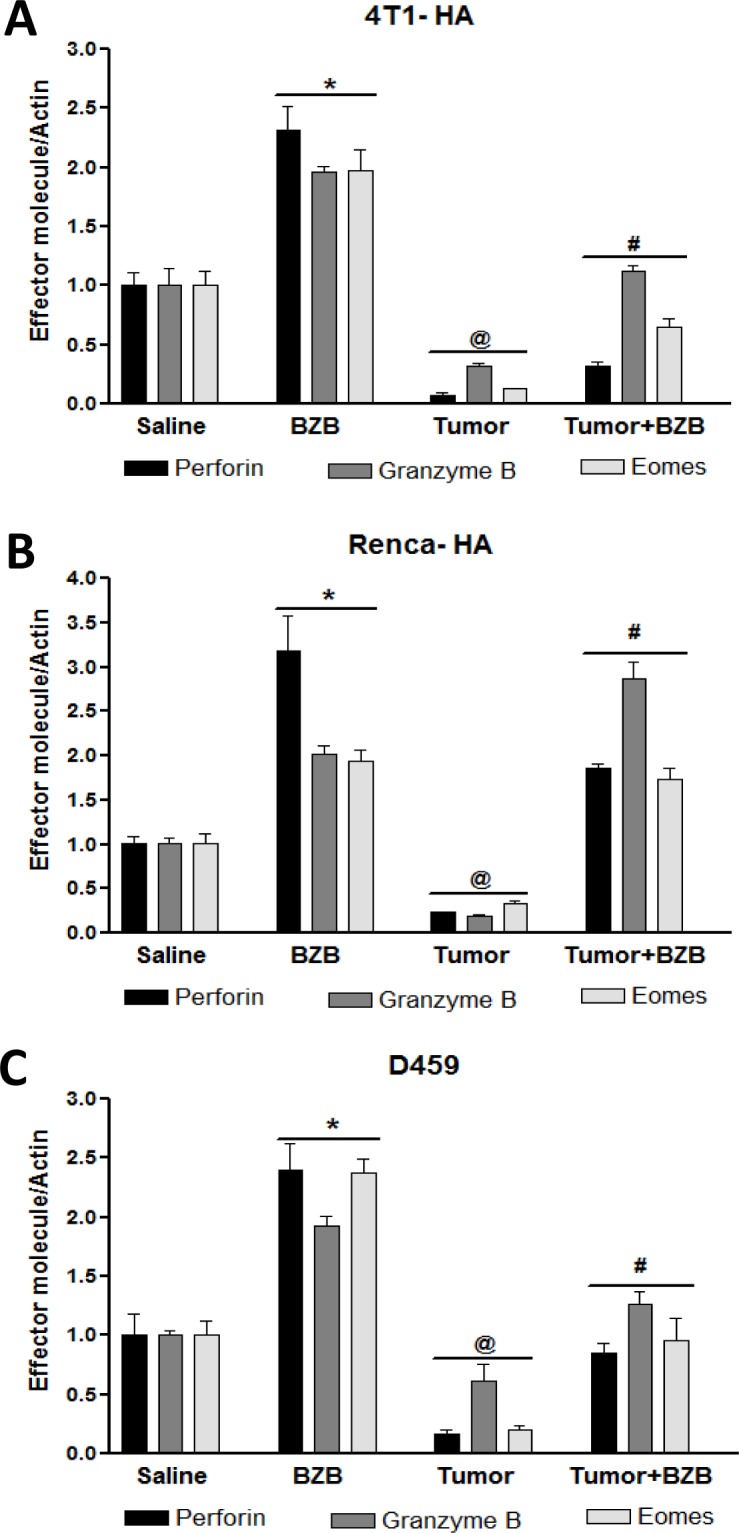
Bortezomib enhances expression of effector molecules in splenocytes of tumor-bearing mice Balb/c WT mice were injected subcutaneously with mammary adenocarcinoma 4T1HA, renal carcinoma RencaHA, or lung fibrosarcoma D459 cells (5 × 10^6^ each) to establish subcutaneous tumors. On day 14, mice were treated intravenously with bortezomib (BZB, 1 mg/kg body weight). Spleens were harvested 4 h after BZB treatment from tumor-bearing or control mice. RBC-depleted splenocytes were analyzed for mRNA expression of effector molecules perforin, granzyme B, and eomesodermin (*Eomes*) by qPCR in 4T1HA **A.**, RencaHA **B.** and D459 **C.** tumor-bearing mice. Data are expressed as mean ± S.E.M; *n* = 4 mice, each group from one representative experiment out of three individual experiments with similar results; *p < 0.05, saline *versus* BZB; ^@^*p* < 0.05, saline *versus* tumor; ^#^*p* < 0.05, tumor *versus* tumor + BZB (ANOVA, one-way).

**Figure 2:**
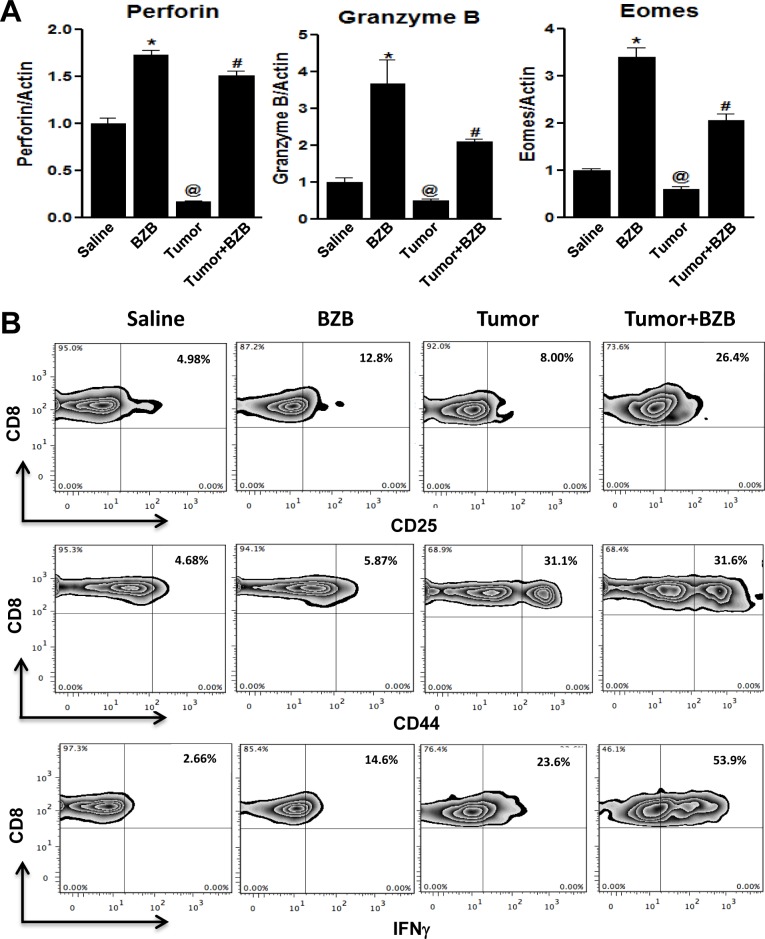
Bortezomib augments expression of CD8^+^ T cell activation and effector molecules in tumor-bearing mice CD8^+^ T cells were purified from the pooled cells of spleens and LN harvested 4 h after bortezomib (1 mg/kg body weight) treatment in Balb/c mice established with 14-day subcutaneous 4T1HA tumors. CD8^+^ T cells were analyzed for mRNA expression of effector molecules perforin, granzyme B and eomesodermin by qPCR **A.**, and for CD25, CD44, and intracellular interferon-γ (IFNγ) on gated CD8^+^ cells by FACS analysis **B.**. Data are expressed as mean ± S.E.M; *n* = 12 mice, each group from three individual experiments; *p < 0.05, saline *versus* BZB; ^@^*p* < 0.05, saline *versus* tumor; ^#^*p* < 0.05, tumor *versus* tumor + BZB (ANOVA, one-way).

Anti-tumor cytolytic activity of T lymphocytes (CTL) is an important determinant for tumor immunosurveillance and lysis. Given conflicting reports implicating suppressive and stimulatory effects of the proteosome inhibitor bortezomib on immune cells [37–44], in this study, we carefully investigated the effects of bortezomib on the effector function of CTLs in tumor-bearing mice. We established subcutaneous tumors of breast adenocarcinoma (4T1HA) and renal carcinoma (RencaHA) that present a defined low-avidity epitope of immunogenic antigen hemagglutinin (HA) [45], as well as lung fibrosarcoma (D459) that expresses human Ras and mutant human p53 as xenogeneic antigens [46], in syngeneic wild type (WT) Balb/c mice for 2 weeks before administering bortezomib intravenously at a tumor therapeutic dose of 1 mg/kg body weight, optimized by us previously [35], which could roughly be correlated to a transient 20 nM concentration on the basis of the observation that a mouse of 20-25 g weight has approximately a blood volume of about 1.5 ml. Four hours after the last bortezomib treatment, single cell suspensions of spleen or lymph nodes (LN) were analyzed. We were intrigued by a reversal of tumor-induced downregulation of effector genes perforin, granzyme B and eomesodermin in the spleens and LN of tumor-bearing mice treated with bortezomib (Figure 1). Similar effect was observed on the expression of effector molecules in purified CD8^+^ T cells from the pooled spleen and LN of 4T1HA tumor-bearing mice treated with bortezomib (Figure 2A), which also showed increased expression of the high-affinity IL-2 receptor α-chain, CD25, along with increased intracellular secretion of IFNγ (Figure 2B). We noted that CD8^+^ T cells following bortezomib treatment in tumor-bearing mice maintained expression of CD44, a late T cell activation marker indicative of effector-memory T cells (Figure 2B).

**Figure 3:**
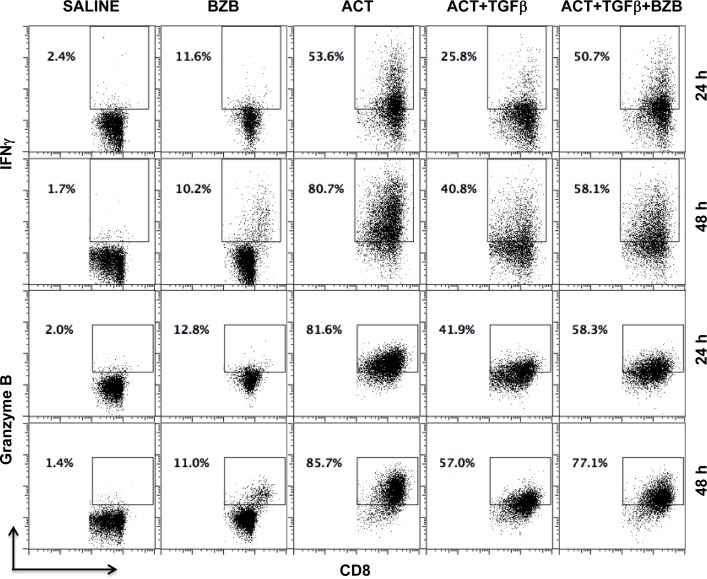
Bortezomib counteracts T cell immunosuppression by TGFβ Purified CD8^+^ T cells from a pool of spleen and lymph node cells from naïve Balb/c mice were stimulated *in vitro* with soluble anti-mouse CD3 and CD28 antibodies (1 μg/ml each) for 24 or 48 h in presence or absence of recombinant mouse TGFβ1 (5 ng/mL) at 37°C followed by treatment with bortezomib (10 nM) for another 4 h. Analysis of intracellular IFNγ and granzyme B by flow cytometry is shown on gated CD8^+^ cells. Representative data are shown from three individual experiments.

We also assessed whether bortezomib can neutralize the suppression of T cell cytolytic molecule expression mediated in the tumor microenvironment by the immunosuppressive cytokine TGFβ *via* the Smad-ATF1 pathway [47]. Purified CD8^+^ T cells were stimulated with CD3 and CD28 antibodies *in vitro* for 24 or 48 h in the presence or absence of TGFβ followed by a 4 h bortezomib treatment. Apparently, TGFβ-mediated suppression of IFNγ and granzyme B expression in activated CD8^+^ T cells was significantly neutralized by bortezomib treatment (Figure 3). These observations suggest that bortezomib treatment has a stimulatory effect on T cell function by enhancing the expression of activation and effector molecules, which has a potential to counteract T cell immunosuppression.

### Bortezomib modulates Notch status in lymphoid organs of tumor-bearing mice

**Figure 4:**
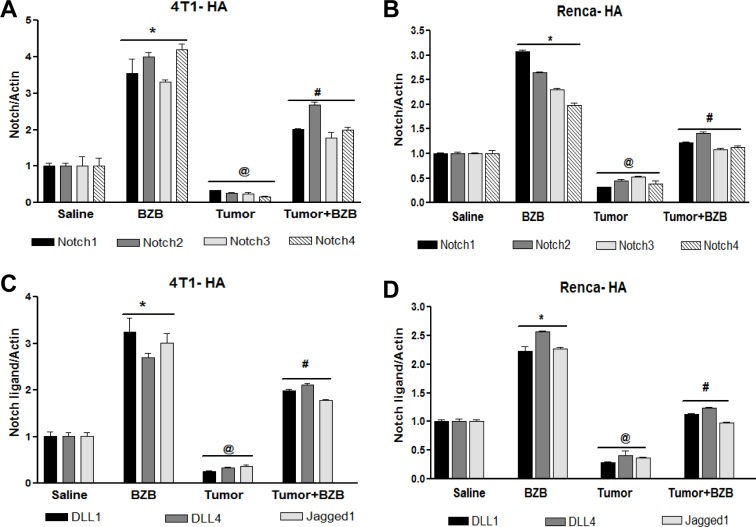
Bortezomib influences Notch system in tumor-bearing mice Splenocytes were harvested 4 h after bortezomib (1 mg/kg body weight) treatment in Balb/c mice established with 14-day subcutaneous tumors. Expression of mRNA is shown for Notch receptors Notch1, Notch2, Notch3, and Notch4 in 4T1HA **A.** and RencaHA **B.**, and for Notch ligands *Dll1*, *Dll4* and *Jag1* in 4T1HA **C.** and RencaHA **D.** tumor-bearing mice. Data are expressed as mean ± S.E.M; *n* = 12 mice, each group; *p < 0.05 saline *versus* BZB, ^@^*p* < 0.05 saline versus tumor, ^#^*p* < 0.05 tumor *versus* tumor + BZB (ANOVA, one-way). Statistical analysis was based on three individual experiments.

**Figure 5:**
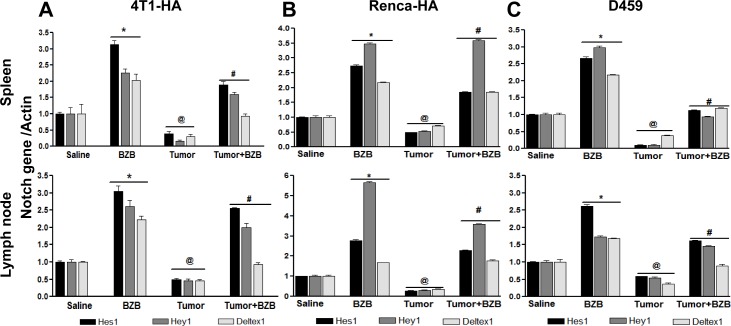
Bortezomib modulates Notch target genes in spleens and lymph nodes of tumor-bearing mice Splenocytes and LN cells were harvested 4 h after bortezomib (1 mg/kg body weight) treatment in Balb/c mice established with 14-day subcutaneous tumors. Expression of mRNA is shown for Notch target genes *Hes1*, *Hey1* and *Deltex1* in splenocytes and lymph node cells of 4T1HA **A.**, RencaHA **B.**, and D459 **C.** tumor-bearing mice. Data are expressed as mean ± S.E.M; *n* = 12 mice, each group; *p < 0.05 saline *versus* BZB, ^@^*p* < 0.05 saline *versus* tumor, ^#^*p* < 0.05 tumor *versus* tumor + BZB (ANOVA, one-way). Statistical analysis was based on three individual experiments.

**Figure 6:**
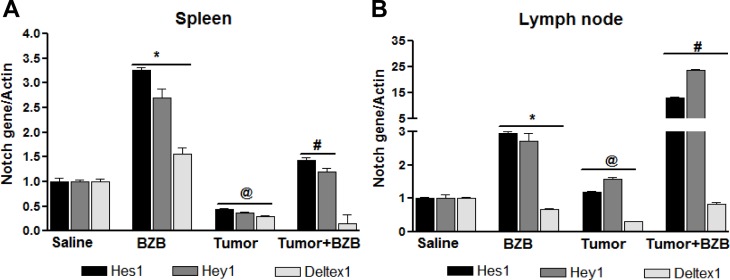
Bortezomib influences Notch system in TCRHA Cln4 tumor-bearing mice TCRHA-transgenic Cln4 mice were injected subcutaneously with mammary adenocarcinoma 4T1HA cells (5 × 10^6^). On day 14, mice were treated intravenously with bortezomib (1 mg/kg body weight). Spleens and LN were harvested from tumor-bearing or control mice 4 h after BZB treatment. Expression of mRNA for *Hes1*, *Hey1* and *Deltex1* was analyzed in RBC-depleted splenocytes **A.** and LN cells **B.**. Data are expressed as mean ± S.E.M; *n* = 4 mice, each group from one representative experiment out of three individual experiments with similar results; *p < 0.05, saline *versus* BZB; ^@^*p* < 0.05, saline *versus* tumor; ^#^*p* < 0.05, tumor *versus* tumor + BZB (ANOVA, one-way).

In an effort to understand the mechanisms underlying the bortezomib-stimulated enhancement of T cell functional molecules, we investigated the effects of the proteasome inhibitor bortezomib on Notch signaling in lymphocytes of tumor-bearing mice. Mice established with subcutaneous tumors of breast 4T1HA, kidney RencaHA, or lung D459 tumor cells for 2 weeks were treated with BZB (1 mg/kg body weight). Four hours after bortezomib treatment, single cell suspensions of spleen, LN and thymus were analyzed. We observed that tumor-induced decrease in the expression of Notch receptors, Notch1, Notch2, Notch3 and Notch4, as well as ligands *Dll1*, *Dll4* and Jagged1 in splenocytes of 4T1HA and RencaHA tumor-bearing mice could be reversed by treatment with bortezomib (Figure 4). Similar results were observed in D459 tumor-bearing mice (data not shown). *Hes1*, *Hey1* and *Deltex1* are the key targets downstream of Notch receptor engagement. Interestingly, bortezomib reversed the tumor-induced downregulation of these Notch cascade genes in the spleen and LN of 4T1HA (Figure 5A), RencaHA (Figure 5B) and D459 (Figure 5C) tumor-bearing mice. We also observed similar effects of bortezomib on Notch cascade genes in the thymus of WT mice bearing different tumor models (Supplementary Figure S1). We further confirmed these results in Cln4 TCR-transgenic mice overexpressing a population of CD8^+^ T cells that reacts specifically to a low-avidity epitope IYSTVASSL on the model antigen hemagglutinin [45] expressed on tumor cells, RencaHA and 4T1HA. TCR-HA Cln4 mice also showed a remarkably increased expression of Notch genes in both spleen and LN following bortezomib treatment (Figure 6). These observations suggest that bortezomib has a significant bearing on the Notch receptor-ligand profile in lymphoid tissues as well as on Notch downstream signaling in immune cells, whereby it can reverse tumor-induced immune suppression.

### Bortezomib administration selectively modulates Notch1/2 signaling in CD8^+^ T cells of tumor-bearing mice

**Figure 7:**
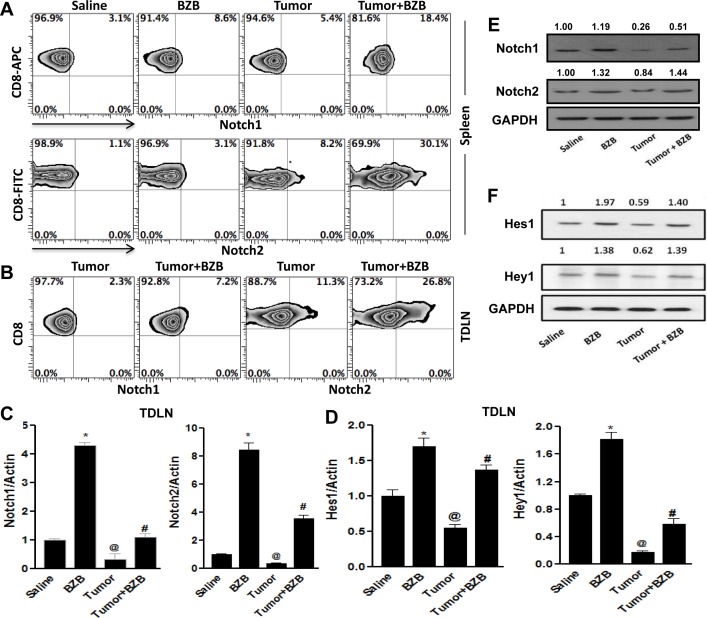
Bortezomib enhances Notch 1/2 signaling in CD8^+^ T cells of tumor-bearing mice CD8^+^ T cells were purified from either the spleens, or tumor-draining LN (TDLN) or a pool of spleens and LN harvested 4 h after bortezomib (1 mg/kg body weight) treatment in Balb/c mice established with 14-day subcutaneous 4T1HA tumors. Purified CD8^+^ T cells were analyzed for the surface protein levels of Notch receptors 1 and 2 by flow cytometry in spleen **A.**, and TDLN **B.**. Expresion of mRNA from purified CD8+ T cells from TDLN were analyzed for Notch1 and Notch2 receptors **C.**, as well as for Notch target genes, *Hes1* and *Hey1* by qPCR **D.**. Protein levels by Western blot were also analyzed in CD8^+^ T cells from a pool of spleens and LN for Notch1 and Notch2 **E.** and Hes1 and Hey1 **F.**. The values on Western blots represent the densitometric quantification. GAPDH was used as a loading control for Western blot. Data are expressed as mean ± S.E.M; *n* = 12 mice, each group; *p < 0.05, saline *versus* BZB, ^@^*p* < 0.05, saline *versus* tumor, ^#^*p* < 0.05, tumor *versus* tumor + BZB (ANOVA, one-way). Statistical analysis was based on three individual experiments.

We next assessed whether the restoration of Notch signaling by bortezomib evident in lymphoid organs of tumor-bearing mice is reflected in the Notch profile of endogenous CD8^+^ T cells, the major cytolytic cells in antigen-specific anti-tumor immune response. Unlike Notch receptor expression in total splenocytes and LN cells (Figure 4), in purified CD8^+^ T cells bortezomib selectively upregulated receptor levels of Notch1 and Notch2 (Figure 7A), with no significant changes in Notch3 and Notch4 receptors (data not shown). This indicated a differential effect by bortezomib on Notch receptors in CD8^+^ T cells. Further, in the tumor-draining LN (TDLN), tumor-specific Vβ8.1/2^+^ CD8^+^ T cells representing a frequency of 8-10% (data not shown) showed increased expression of the surface receptor and mRNA of Notch1 and Notch2 following bortezomib treatment (Figure 7B and 7C). Analysis of the expression of downstream Notch target genes *Hes1* and *Hey1* in the TDLN showed similar effect (Figure 7D). We also analyzed the post-transcriptional protein levels of Notch receptors and target gene products in CD8^+^ T cells purified from the pooled spleen and LN of control and 4T1HA tumor-bearing WT mice treated with bortezomib. A significant upregulation of protein levels of Notch1 and Notch2 receptors (Figure 7E), and Hes1 and Hey1 products (Figure 7F) was observed in bortezomib-treated group when compared with tumor alone group. These observations suggest that bortezomib can impact CD8^+^ T cell function by enhancing their Notch1/2 receptor signaling.

### Bortezomib facilitates crosstalk between NICD and NFκB activity

**Figure 8:**
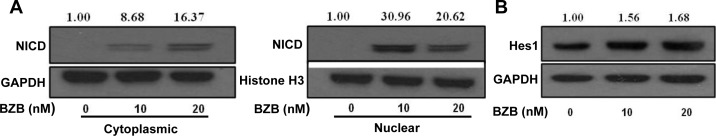
Bortezomib upregulates the expression of NICD and its downstream target Hes1 in CD8^+^ T cells CD8^+^ T cells purified from pooled cells of spleen and LN from naïve Balb/c mice were treated with 10 or 20 nM BZB for 4 h. Their nuclear and cytoplasmic extracts were analyzed for cleaved Notch intracellular domain (NICD) expression **A.**, and total protein extract was analyzed for Hes1 expression **B.** by Western blots. The values represent the densitometric quantification of the immunoblots. GAPDH and Histone H3 were used as loading controls.

**Figure 9:**
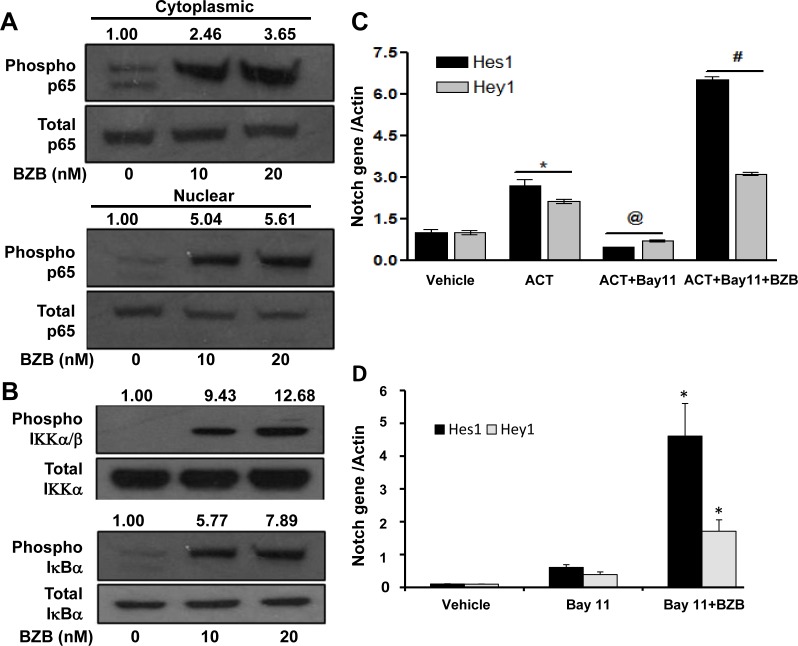
Bortezomib stimulates the expression of Notch genes *via* NFκB activation CD8^+^ T cells purified from pooled cells of spleen and LN from naïve Balb/c mice were treated with 10 or 20 nM BZB for 4 h. **A.** Total and phosphorylated forms of p65 analyzed in the nuclear and cytoplasmic extracts, and **B.** total and phosphorylated forms of IKK and IκBα analyzed in whole cell preparations of BZB-treated CD8^+^ T cells by Western blots. The values represent the densitometric quantification of the immunoblots. GAPDH was used as loading control. Expression of *Hes1* and *Hey1* mRNA in CD8^+^ T cells stimulated *in vitro* using CD3 and CD28 antibodies (1 μg/ml, 24 h) **C.**, or naïve without stimulation **D.**, followed by treatment with BZB (10 nM, 4 h) along with NFκB inhibitor Bay-11-7082 (5 μM). Data are expressed as mean ± S.E.M; *n* = 3. *p < 0.05, vehicle versus activation or Bay11 *versus* Bay11 + BZB; ^@^*p* < 0.05, activation (ACT) *versus* ACT + Bay11; ^#^*p* < 0.05, ACT + Bay11 versus ACT + Bay11 + BZB (ANOVA, one-way). Statistical analysis was based on one representative experiment out of three individual experiments.

We next investigated the mechanisms of how bortezomib activates Notch and causes upregulation of effector molecules in T cells. Two downstream pathways of Notch signaling that can affect T cell function are mediated *via* Notch intracellular domain and NFκB. We tested the roles of these pathways in purified CD8^+^ T cells that were treated *in vitro* with bortezomib at different doses (10-20 nM) for 4 h similar to our *in vivo* treatment regimen. Results show that bortezomib treatment upregulated the protein expression of NICD in the cytoplasmic fraction of CD8^+^ T cells in a dose-dependent manner, with a pronounced increase in the nuclear levels of NICD (Figure 8A). We are not clear whether there is any contribution of nuclear translocation of NICD in the observed increase in the nuclear fraction of NICD. Increased Hes1 protein was also observed in CD8^+^ T cells following bortezomib treatment (Figure 8B). We then assessed the role of NFκB activation in bortezomib-induced effects on Notch signaling. Treatment of CD8^+^ T cells from naïve mice with bortezomib (10 or 20 nM; 4h) showed a dose-dependent increase in the phosphorylation of NFκB p65 in both nuclear and cytoplasmic fractions (Figure 9A) as well as an induction of IKKα/β and IκBα phosphorylation (Figure 9B). The IKK kinase complex is the core element of the NFκB cascade, essentially made of two kinases (IKKα and IKKβ) and a regulatory subunit, NEMO/IKKγ. Depending on the activating signal and the cell type, NFκB activation can be mediated by the canonical IKKβ and NEMO, or the noncanonical IKKα pathways. Although from our data it is not clear which IKK subunit is involved, a clear IκBα phosphorylation and nuclear upregulation of p65, thus suggesting enhanced NFκB activation, is evident. To further dissect the mechanism, we tested the effect of bortezomib (10 nM; 4 h) on CD8^+^ T cells that were pre-incubated for 24 h with a compound Bay-11-7082 (Bay 11), which is an irreversible inhibitor of IKKα, with or without CD3 and CD28 agonist antibodies. Results show that both *Hes1* and *Hey1* mRNA were significantly decreased in CD8^+^ T cells when activated in presence of Bay 11, confirming the role of NFκB in Notch signaling (Figure 9C). Moreover, treatment with bortezomib significantly abrogated the effect of Bay 11. We observed a remarkably increased expression of *Hes1* and *Hey1* following bortezomib treatment in presence of Bay 11 both in activated CD8^+^ T cells as well as in naïve CD8^+^ T cells (Figure 9C and 9D). These results suggest that bortezomib has an intrinsic ability to increase the levels of NICD and phosphorylation of intermediaries such as IKK, IκBα and p65 in CD8^+^ T cells, thereby facilitating a crosstalk between NICD and NFκB activity.

### Bortezomib-mediated enhancement of effector molecules in T cells is linked to Notch signaling

**Figure 10:**
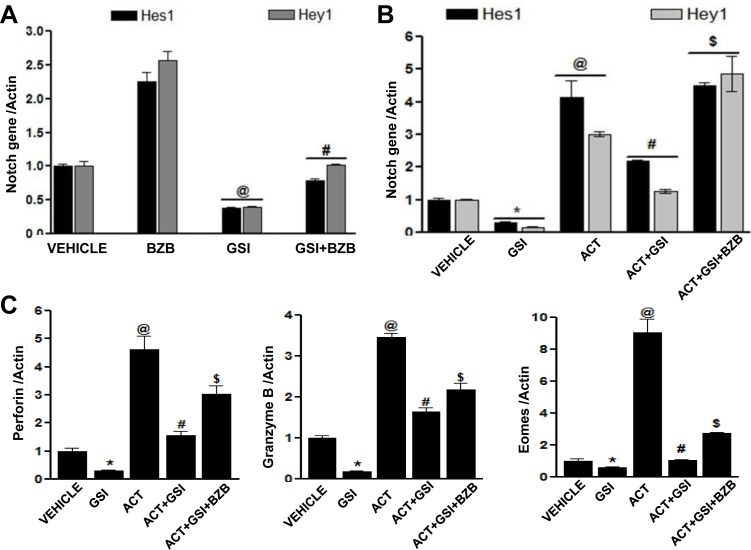
Bortezomib-mediated effect on T cell effector molecules is linked to Notch signaling Lymph node cells from WT Balb/c mice established with 14-day subcutaneous 4T1HA tumors were pre-treated with GSI (500 nM) for 1 h, followed by BZB (10 nM) treatment for 4 h. Data show expression of *Hes1* and *Hey1* mRNA **A.** as mean ± S.E.M; *n* = 12 pooled from 3 independent experiments; ^@^*p* < 0.05, vehicle *versus* GSI; ^#^*p* < 0.05, GSI *versus* BZB + GSI (ANOVA, one-way). Expression of mRNA for *Hes1* and *Hey1* **B.** and effector molecules perforin, granzyme B and eomesodermin **C.** in purified CD8^+^ T cells (from the pooled splenocytes and LN cells of WT Balb/c mice) pre-treated with GSI (500 nM) for 1 h and activated with CD3 and CD28 antibodies (1 μg/ml) for 24 h, followed by BZB (10 nM) treatment for 4 h. Data are expressed as mean ± S.E.M; *n* = 3; *p < 0.05, vehicle *versus* GSI; ^@^*p* < 0.05, vehicle *versus* activation; ^#^*p* < 0.05, activation *versus* activation + GSI; and ^$^p<0.05, activation + GSI *versus* activation + GSI + BZB (ANOVA, one-way). Statistical analysis was based on three individual experiments.

To further understand the mechanisms underlying bortezomib's influence on Notch activation and enhancement of effector molecules in T cells, we performed an experiment in which LN cells were isolated from WT mice, which were established with 4T1HA tumors for 2 weeks and where an endogenous HA-specific T cell response was expected to be going on. The LN cells *ex vivo* were pre-treated with γ-secretase inhibitor (GSI; 500 nM) for 1 h, before treating with bortezomib (10 nM) for another 4 h. Analysis of mRNA expression in LN cells revealed that bortezomib significantly increased the levels of Notch genes *Hes1* and *Hey1* even in the presence of GSI (Figure 10A). We also evaluated the cell-specific effect of bortezomib treatment on Notch genes and T cell effector molecules in an *in vitro* set-up where we stimulated purified CD8^+^ T cells from naïve mice with CD3 and CD28 antibodies (1μg/ml) for 24 h in presence of γ-secretase inhibitor (GSI; 500 nM) followed by treatment with bortezomib (10 nM) for 4 h. Indeed, bortezomib upregulated mRNA expression of Notch *Hes1* and *Hey1* genes as well as eomesodermin, granzyme B and perforin in CD3/CD28-stimulated CD8^+^ T cells in presence of GSI, while treatment with GSI alone significantly reduced the expression of these molecules (Figure 10B and 10C). Noticeably, bortezomib was able to upregulate the expression of T cell effector molecules in the presence of GSI, albeit not to the untreated levels.

**Figure 11:**
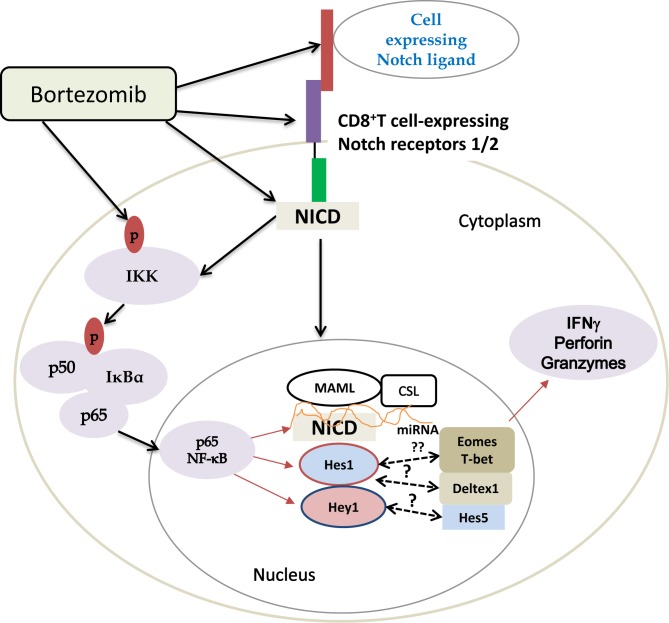
Scheme depicting bortezomib's influence on Notch-NFκB crosstalk affecting expression of T cell effector molecules Bortezomib increases the levels of cleaved Notch intracellular domain and phosphorylation of NFκB p65. Translocated p65 in the nucleus may interact with Notch components, thereby activating Notch target genes and T-box transcription factors, which subsequently improve the expression of T cell effector molecules in tumor-bearing mice.

Altogether, these results, schematically presented in Figure 11, demonstrate that bortezomib treatment has a stimulatory effect on T cell effector function. This is mediated by elevated Notch1/2 receptor expression (Figure 7) and downstream Notch-NFκB crosstalk following bortezomib's direct effects on the accumulation of cytoplasmic and nuclear NICD (Figure 8), increased phosphorylation of IKKs, IκBα, and p65 (Figure 9) along with increased expression of Notch genes *Hes1* and *Hey1* and T-box transcription factors eomesodermin (Figure 2, 6 and 9) and T-bet (data not shown). Thus, it is clear that a low-dose bortezomib treatment tested in this study can enhance anti-tumor CD8^+^ T cell function by promoting Notch-NFκB crosstalk, which has a potential to neutralize T cell immunosuppression by tumor.

## DISCUSSION

Tumor-induced immunosuppression is a major hurdle associated with cancer that renders immunotherapy inefficient. It is therefore essential that efforts at achieving effective immunotherapy take into consideration the need to address immune suppression by tumor. Notch receptors and ligands are expressed in developing lymphoid tissue and control both functional differentiation and maturation of T cells [48–50]. Evidence has accumulated to show that Notch regulates effector function of T cells [26, 51, 52] though the mechanisms are unclear. Thus, agents that can selectively enhance Notch signaling in anti-tumor immune cells are much needed. Results of our study show that bortezomib can be a promising candidate for modulating the Notch system to enhance anti-tumor immune responses. Bortezomib is a dipeptide boronate proteasome inhibitor that blocks intracellular protein turn-over. Studies have shown that bortezomib could sensitize not only myeloma and lymphoma cells, but also a variety of human and mouse solid tumor cells to apoptosis [27, 28, 35, 36], thus providing an exciting opportunity for the development of novel anticancer therapeutics [53]. Bortezomib has also been reported to affect several cellular pathways including NFκB, histone deacetylases (HDACs) and death receptor signaling in tumor cells [54–56]. However, bortezomib's influence on anti-tumor immune responses has been controversial. We, therefore, investigated the effects of BZB on the expression of effector molecules and Notch-NFκB pathways in T cells using a tumor therapeutic dose of bortezomib optimized by us previously [35].

We found that lung cancer patients show impaired Notch signaling in the bone marrow as well as the secondary tissues of the immune system such as thymus, spleen and lymph node [26]. Tumor-induced dysregulation of Notch was confirmed in mice bearing tumor models such as breast adenocarcinoma 4T1HA, renal carcinoma RencaHA and lung fibrosarcoma D459, which showed downregulation of Notch receptors, Notch1, Notch2, Notch3 and Notch4 as well as Notch ligands, *Dll1*, *Dll4* and Jagged1 in their lymphoid organs. Interestingly, administration of bortezomib to tumor-bearing mice restored Notch components in these organs to levels either more or the same as observed in untreated naïve mice. We observed that bortezomib increased the expression of *Dll1* in lymphoid tissues as well as its cognate receptors Notch1 and Notch2 on CD8^+^ T cells. We also noted that bortezomib increased the expression of *Dll4* and Jagged1 in tested lymphoid tissues, which are reported to play a role in regulatory T cell differentiation. However, we observed no significant changes in regulatory T cell populations following bortezomib treatment (data not shown). These effects of bortezomib warrant further investigation. Here, we focused on dissecting the effects of bortezomib on Notch activation in anti-tumor CD8^+^ T cells and their relevance for T cell activation and effector function. Primary Notch targets include two families of transcriptional repressors, hairy and enhancer-of-split-related basic helix-loop-helix (bHLH) such as *Hes* and *Hey*. *Deltex1* is another known Notch gene that is considered a transcriptional target of nuclear factor of activated T cells (NFAT) to promote T cell anergy [57]. We show that bortezomib administration to naïve or tumor-bearing mice upregulates mRNA expression of *Hes1* and *Hey1* and their protein products. However, no major change in *Deltex1* expression was observed. Thus, bortezomib appears to enhance Notch activation in CD8^+^ T cells *via* upregulation of Notch1 and Notch 2 and their downstream *Hes1* and *Hey1* genes.

Notch1 has been reported to bind to the promoters of T-box transcription factor eomesodermin as well as perforin and granzyme B [51] that mediate the effector function of T cells [58]. However, mechanisms are not clear as to how therapeutic activation of Notch can influence the CD8^+^ T cell function to promote anti-tumor immune responses. In this study, we observed that CD8^+^ T cells in tumor-bearing mice following bortezomib administration not only showed increased Notch1/2 activation but also sustained increased expression of T cell activation molecules CD25 and CD44, with significantly improved production of IFNγ and expression of eomesodermin, perforin and granzyme B. Eomesodermin is not only important for the differentiation of cytolytic CD8^+^ T cells but also helps maintain memory CD8^+^ T cell repertoire [59]. We also noted that bortezomib treatment upregulated the expression of T-bet, another T-box transcription factor (data not shown), which plays a key role in cytolytic T cell immunity. Bortezomib, thus, can not only enhance T cell cytolytic activity but may also help in the maintenance of the memory T cell pool.

On examining direct effects of bortezomib on the canonical Notch signaling in CD8^+^ T cells, we found that bortezomib increased the cleaved free Notch1 intracellular domain in a dose dependent manner in both cytoplasmic and nuclear fractions, and, as expected, increased the levels of its downstream targets *Hes1* and *Hey1*, even in presence of the γ-secretase inhibitor, compound E that blocks the NICD cleavage at the S3 site. This suggests that bortezomib has an intrinsic ability to regulate intramembrane Notch proteolysis, or retention of cleaved NICD, either by (a) augmenting ADAM10-mediated membrane proximal cleavage at S2 site, thereby providing more S2 substrates, which are short-lived and rate-limiting, for subsequent γ-secretase cleavage at S3, or (b) allowing Notch cleavage at a site downstream of γ-secretase activity, and/or (c) enhancing nuclear localization of NICD. NICD has also been reported to directly interact with NFκB to affect transcriptional activity of nuclear NFκB [19].

NFκB is released from the cytoplasm following the phosphorylation of IκB kinase complex that induces proteasomal degradation of IκBα, enabling subunits of the active NFκB to translocate to the nucleus to induce target gene expression and IFNγ production [60]. We observed that inhibition of NFκB activity in CD8^+^ T cells by Bay-11-7082, which blocks IKKα, downregulated their expression of Notch genes *Hes1* and *Hey1*, underlining an important crosstalk between Notch and NFκB pathways. Data show that bortezomib enhances NFκB activity in CD8^+^ T cells, in contrast to multiple myeloma cells where bortezomib was shown to inhibit NFκB activity by preventing proteosomal degradation of IκBα [61]. One other study demonstrated differential effects of bortezomib on NFκB activity depending on different cell types: bortezomib triggered NFκB activity *via* canonical pathway in peripheral blood mononuclear cells by non-proteosomal degradation of IκBα while NFκB activation was inhibited in bone marrow-derived stromal cells [54]. We observed a dose-dependent increase in the phosphorylation of nuclear and cytoplasmic p65 as well as IκBα and IKK in CD8^+^ T cells following bortezomib treatment, with no inhibition by Bay 11, confirming bortezomib-mediated stimulatory effects on NFκB activation. It is also possible that bortezomib augmented the activation and nuclear translocation of other transcription factors such as the activator protein 1 (AP-1), a heterodimer of c-Fos and c-Jun, which is activated downstream of the ERK and JNK pathway. It will be interesting to further investigate the effects of bortezomib on other CSL (RBP-Jκ in mice)-independent Notch signaling pathways, such as Hedgehog, Jak/STAT, PI3K/Akt, mTOR, and MEK/ERK, which are also implicated in non-canonical Notch signaling [62–65].

Collectively, results show that bortezomib can restore Notch signaling in anti-tumor CD8^+^ T cells by enhancing a crosstalk between Notch and NFκB pathways *via* its multiple effects on the expression of Notch1 and Notch2 receptors, NICD, and downstream Notch genes, together with increased phosphorylation of the IκB kinase, IκBα and p65, which subsequently stimulate NFκB activity. Consequently, this bortezomib-enhanced Notch-NFκB crosstalk improves anti-tumor cytolytic functions such as IFNγ production and expression of effector molecules by CD8^+^ T cells. We are currently exploring post-transcriptional regulation of these effects by bortezomib at the level of small non-coding microRNAs in CD8^+^ T cells as well as the relevance of bortezomib-mediated T cell effects in various immunotherapeutic settings. Findings of this study predict that bortezomib can act as a safe and potent immunotherapeutic drug in sustaining anti-tumor immune effector functions and in addressing tumor-associated immunosuppression if well optimized in conjunction with other immune therapies.

## MATERIALS AND METHODS

### Mice

Balb/c mice (6-8 week old) were purchased from Harlan (Indianapolis, IN). TCRHA-transgenic Cln4 mice were provided by Linda A. Sherman, The Scripps Research Institute, La Jolla, CA, and were bred at Meharry Medical College animal facility and genotyped according to the methods described earlier [45]. Mice were cared for in accordance with the procedures outlined in the National Institutes of Health *Guide for the Care and Use of Laboratory Animals.* Meharry Medical College is accredited by the Association for Assessment and Accreditation of Laboratory Animal Care International and follows the Public Health Service Policy for the care and use of laboratory animals under pathogen-free conditions.

### Tumor cell lines

The RencaHA line (courtesy Hyam I. Levitsky, John Hopkins University, Baltimore, MD), 4T1 (courtesy Suzanne Ostrand-Rosenberg, University of Maryland, Baltimore, MD), and D459 were maintained in FCS-supplemented standard RPMI-1640 culture medium. D459 cells are murine fibroblasts malignantly transformed by transfection of human *Ras* and mutant human *p53* [46]. Low-passage (< 5) tumor cell cultures were used for the experiments.

### Solid tumor induction and tissue harvest

Solid tumors were induced in syngeneic Balb/c WT or Cln4 mice by injecting 4T1HA, RencaHA or D459 cells subcutaneously at the indicated cell numbers for at least 10 days. Following the establishment of palpable subcutaneous tumors of approximately 120 mm^3^ size, mice were injected with bortezomib (1 mg/kg body weight) intravenously and 4 h later lymphoid tissues or tumor mass were harvested for the preparation of single cell suspensions.

### CD8^+^ T cell purification and activation

CD8^+^ T cells were purified from pooled spleen and lymph node cells by incubating cells with rat anti-mouse CD8 mAb, followed by positive selection of CD8^+^ cells with anti-rat IgG Microbeads (Miltenyi Biotec). For experiments using GSI *in vitro*, purified CD8^+^ T cells were pretreated with GSI (500 nM; R&D Systems) for 30 min at 37°C or with DMSO as vehicle control and then stimulated with soluble anti-mouse CD3 and CD28 antibodies (1 μg/ml each; Biolegends) for 24 h, followed by treatment with bortezomib (10 nM) for another 4 h. Similarly, for NFκB inhibition experiments, CD8^+^ T cells were pretreated with 5 μM Bay 11-7082 (Sigma) before stimulation with CD3 and CD28 antibodies and treatment with bortezomib.

### TGFβ suppression

For experiments using TGFβ *in vitro*, purified CD8^+^ T cells from pooled spleen and lymph node cells from naïve Balb/c mice were stimulated *in vitro* with soluble anti-mouse CD3 and CD28 antibodies (1 μg/ml each; Biolegends) for 24 or 48 h in presence or absence of treatment with 5 ng/mL recombinant mouse TGFβ (R&D Systems) at 37°C followed by treatment with bortezomib (10 nM) for another 4 h. TGFβ was constituted in a buffer comprising 1N HCL and fetal calf serum. At the end of the culture, cells were analyzed for IFNγ and granzyme B secretion by flow cytometry.

### RNA isolation and quantitative PCR

Total RNA was extracted using an RNeasy mini kit (Qiagen) and quantitated by reading the optical density at 260 nm. Possible genomic DNA contamination was removed by on-column DNase digestion using the RNase-free DNase set. The cDNA was synthesized using iScript cDNA synthesis kit (Bio-rad). Real-time quantitative RT-PCR (qRT-PCR) was performed using CFX-96 Real Time System (Bio-rad). The iQ SYBR green supermix (Bio-rad) and gene-specific PCR primers as listed in Supplementary Table 1 were used in a 20 μL reaction following protocols recommended by the manufacturer. The conditions used for the PCR were as follows: 95°C for 3 min (1 cycle), 94°C for 20 s, 55°C for 30 s, and 72°C for 40 s (40 cycles). Fold changes in mRNA expression were assessed by the ΔΔCt method.

### Flow cytometry

Single cell suspensions of RBC-depleted splenocytes and lymph node cells (2 × 10^5^ cells) were stained for surface or intracellular immunofluorescence staining with various antibodies or control isotypes (BD Biosciences, San Jose, or eBioscience Inc., San Diego, CA) following FcγR-blocking. For surface staining, cells were incubated with fluorochrome-conjugated mAbs in the dark on ice for 30 min. IFNγ intracellular staining was measured in T cells following *ex vivo* re-stimulation for 5 h with 200 ng/ml ionomycin plus 10 ng/ml phorbol 12-myristate 13-acetate (Sigma, St. Louis, MO) in the presence of GolgiStop^TM^ protein transport inhibitor (BD Biosciences) as per manufacturer's protocol. Briefly, cells were permeabilized for 20 min with fixation/permeabilization kit (BD Cytofix/Cytoperm™) prior to staining with intracellular antibodies. Data were acquired using a Guava EasyCyte HT system (EMD Millipore) or a FACS Calibur (BD Biosciences) and analyzed by FlowJo software (Treestar Inc.). Following monoclonal antibodies were used: CD8α-FITC, CD8α-PE, Vβ8.1/2-PE, Vβ8.1/2-FITC, CD44-FITC, CD25-PE, IFNγ-PE, granzyme B-PE, Notch1-APC, and Notch2-PE.

### Immunoblotting

CD8^+^ T cells from tumor-bearing or naïve mice were purified as mentioned earlier and cell pellets were lysed in complete lysis buffer including protease and phosphatase inhibitors. Nuclear and cytoplasmic extracts were prepared using Ne-per nuclear and cytoplasmic extraction reagent (Life Technologies). Fifty micrograms of each protein sample was electrophoresed on NuPage 4-12% Bis-Tris gel (Novex Life Technologies) and transferred to polyvinylidenedifluoride membranes using an iBlot® Dry Blotting system (Life Technologies). The membrane was then blocked in 5% skimmed milk in 1X PBS-Tween-20 (1X PBST) for 2 h at room temperature with gentle agitation. After blocking, the blots were incubated with rabbit anti-mouse antibodies against Hes1, Hey1 (1:1000; abcam, MA), NICD (Val1744), total and phosphorylated protein of p65 (total D14E12; phospho-S468), IKKα, phospho-IKKα/β (S176/180) and IκBα (total L35A5; phospho-S32/36) (Cell Signaling Technologies) in 1% BSA (in 1X PBST) overnight at 4°C with gentle agitation. After five washes of 5 minutes each in 1X PBST, blots were then incubated with goat anti-rabbit horseradish peroxidase (Santa Cruz Biotechnology) at a dilution of 1:4000 in 1X PBST for 1.5 h, with agitation. The blots were rinsed again in 1X PBST, and developed by using chemiluminescence reagent (EMD Millipore, MA) and a Kodak Image Station. The density of each protein band was determined by densitometric analysis using the imageJ software (NIH). The blots were then stripped and probed with HRP conjugated anti-GAPDH antibody (Cell Signaling Technologies) to determine equivalent loading.

### Statistics

Data were obtained from at least three independent experiments and are presented as means ± S.E.M. Comparisons of mean values between the groups were analyzed using GraphPad Prism 5. Statistical significance of the differences was analyzed by applying two-tailed paired Student *t*-test or by analysis of variance (ANOVA), with p-values < 0.05 considered statistically significant.

## SUPPLEMENTARY MATERIAL FIGURE AND TABLE


