# Supplementary material for: Bortezomib enhances expression of effector molecules in anti-tumor CD8+ T lymphocytes by promoting Notch-nuclear factor-κB crosstalk
Source: Oncotarget. 2015 Sep 28;6(32):32439–55. doi: 10.18632/oncotarget.5857 (PMC4741704; doi:10.18632/oncotarget.5857)
Supplement: Supplementary file 1 [file oncotarget-06-32439-s001.pdf]

Bortezomib enhances expression of effector molecules in anti-tumor CD8+ T lymphocytes by promoting Notch-nuclear factor-κB crosstalk

Supplementary Material

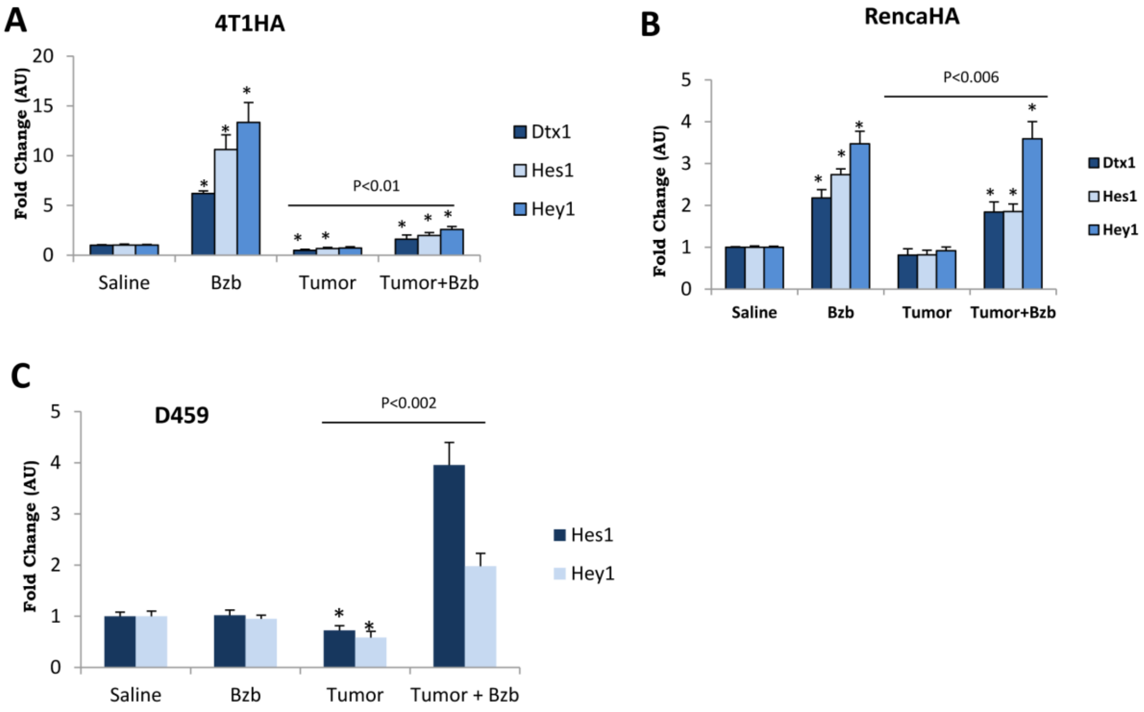

Supplementary Figure 1. Effect of bortezomib on Notch genes in the thymus of tumor-bearing mice

Thymocytes were harvested 4 h after bortezomib (1 mg/kg body weight, i.v.) treatment in Balb/c mice established with 14-day subcutaneous tumors of 4T1HA, RencaHA and D459. Expression of mRNA is shown for Notch target genes *Hes1*, *Hey1* and *Deltex1* in thymocytes of 4T1HA **(A)**, RencaHA **(B)**, and D459 (**C**) tumor-bearing mice. Data are expressed as mean ± S.E.M; n = 12 mice, each group; three individual experiments; *p<0.05 (ANOVA, one-way). All treated groups were compared to the saline administered mice.

Supplementary Table 1. Gene-specific PCR primers.

Target	Forward primer 5′-3′	Reverse primer 5′-3′
Notch1	ACC CAC TCT GTC TCC CAC AC	GCT TCC TTG CTA CCA CAA GC
Notch2	TCG ATG ACT GTG CCT ATG CT	AAA TGT ACT GCC CGT TCA GG
Notch3	GTG TCA ATG GTG GTG TCT GC	CTC ACA CAA AGT GCC CTC AA
Notch4	CTG ACC CCT CAC TTC TCC TG	ACT GGA TCT GGG GGT ATG TG
Hes1	GCC AAT TTG CCT TTC TCA TC	AGC CAC TGG AAG GTG ACA CT
Hey1	CTC TCA GCC TTC CCC TTT TC	ATC TCT GTC CCC CAA GGT CT
Deltex1	TCC GCA TCG TCT ATG ACA TC	AAC CAA ACT CCG TCT TGT GG
Jag1	GGG AAC CCT GTC AAG GAA AT	GGA CGC CTC TGA ACT CTG AC
Jag2	CGT GGC TGC TAT CAC TCA GA	AGC CAC AGC ACA CTG AAC AC
DLL1	CCG GCT GAA GCT ACA GAA AC	AGC CCC AAT GAT GCT AAC AG
DLL4	GCA GCT GTA AGG ACC AGG AG	ATT CGC AGG CAT AAC TGG AC
Perforin	TCT TGG TGG GAC TTC AGC TTT	TGC TTG CAT TCT GAC CGA GT
Granzyme	ACA AAG GCA GGG GAG ATC AT	CGA ATA AGG AAG CCC CCA CA
Eomes	GAA CTC AGA GCT AAG TCC TCT TC	CTT CCT TCC TTC CTT CCT TCT T
Murine β-actin	AGT GTG ACG TTG ACA TCC GTA	GCC AGA GCA GTA ATC TCC TTC
